# Supplementary material for: Effects of Er: YAG laser and acid etching on bond strength of clear aligner attachments to fluorotic enamel
Source: PLoS One. 2025 Aug 7;20(8):e0328937. doi: 10.1371/journal.pone.0328937 (PMC12331112; doi:10.1371/journal.pone.0328937)
Supplement: Attachment — Submitted filename: review end.docx [file pone.0328937.s004.docx]

-The paper can be suitable to acceptance and published if make some revision. The first one about the title .A slightly more detailed version could be:

"Effects of Er:YAG Laser and Acid Etching on Bond Strength of Clear Aligner Attachments to Fluorotic Enamel"

Also the **suggested keywords**:

Dental fluorosis, Orthodontic bracket ,Clear aligner attachment,Shear bond strength,Er:YAG laser,Acid etching,Surface treatment.

**Abstract**

There are some points to enhance the abstract
**a-Too Much Detail:**The abstract includes excessive technical details (e.g., fracture modes, P-values); focus on main findings.

**b-Language/Grammar Issues:**Some awkward phrasing and subject-verb agreement errors; cleaner, professional language is needed.
**c-Abstract Structure:**The structure is generally fine but would benefit from clearer sections (Background, Methods, Results, Conclusion).I**t must** Tighten content and improve flow.

1 Introduction

There are some points about introduction

**a-Excessive Review Detail:**
The review overemphasizes past laser studies, particularly Er:YAG use in periodontitis and bracket rebonding (lines 62–73).**Suggestion:**Streamline the review to focus on bond strength; shift unrelated topics to the Discussion if needed.

b- **Organization and Flow:**
Transitions between paragraphs are abrupt, jumping from fluorosis prevalence to laser effects without clear links.**Suggestion:** Add linking sentences to guide readers from the problem to current solutions, gaps, and study aim.

c-**Study Aim Needs Sharpening:**
The aim is present but should be stated more clearly and placed earlier—ideally by the end of the first or second paragraph.
 **Current Aim:** “Therefore, the purpose of this study was to investigate whether combining Er:YAG laser and acid etching could improve the bonding strength of clear aligner attachments on fluorotic enamel compared to conventional acid etching alone.”

**2 Materials and Methods**

**2.1 Sample collection**

The sample collection is ethical and appropriate but needs clearer reporting on sample size, randomization, and storage
a-Sample Size Justification Missing:No explanation for selecting 20 healthy and 90 fluorotic teeth.

Suggestion: Add justification (e.g., power analysis, precedent studies).
b-Randomization Not Fully Explained: Randomization method is unclear.
Suggestion: Briefly describe how randomization was performed.
c-Storage Method Could Be Clearer: Sample storage is mentioned but lacks detail on preservation quality.
Suggestion: State how specimen integrity was maintained.

**2.2 Sample preparation**

The sample preparation is clear, replicable, and professionally presented, but would be even stronger if it included full laser details, minor clarifications about the template, and a note on blinding.

a- Laser Parameters Missing Details:Laser settings are provided but lack specifics like pulse duration or energy density.
 Suggestion: Include full laser specifications for reproducibility.
b-Attachment Template Clarification Needed:The mention of "Clear aligner, Invisalign" could confuse readers.
Suggestion: Clarify that a custom template was used, not actual Invisalign aligners.
c-Blinding Not Mentioned:It’s unclear whether the SBS measurement operator was blinded.
Suggestion: Mention blinding or explain its absence.

**2.3 Observation of enamel surface structure by Scanning Electron Microscope (SEM)**

The SEM methodology supports the study goals well and is technically adequate, but it needs clearer reporting of magnifications, sample numbers, selection method, and observer blinding

a- Magnification Range Needs Clarification:Magnification range (×3000 to ×50,000) is given, but which was used for each sample image?
 Suggestion: Specify magnification per group or surface type for clarity.
b- Specimen Selection Not Explained:Random selection of specimens is mentioned, but the method isn’t detailed.
Suggestion: Explain the randomization method for transparency.
c-Replication/Number of Samples Not Stated:It’s unclear how many samples were imaged per group.
Suggestion: State the number of specimens per group to show replication.
d-Blinding Not Mentioned:No mention if the SEM observer was blinded.
Suggestion: Specify if blinding was used to reduce bias.

**2.4 Determination of Shear Bond Strength (SBS)**

The SBS method is sound but requires technical clarifications (embedding, test conditions, blinding).

a-Sample Embedding Details Missing:No details on embedding mold, alignment, or material setting.
Suggestion: Describe sample alignment and use of molds for standardization.
b- Test Environment Not Mentioned:It’s unclear whether the SBS test was done dry or wet.

Suggestion: Specify test conditions (dry or wet) for clarity.
c-Blinding Not Addressed:No mention of blinding during SBS measurement.
Suggestion: Indicate if the tester was blinded to reduce bias.
d-Bonding Area Precision:Bonding area measurements are given but not verified post-curing.
Suggestion: State if bonding area was checked after curing.

**2.5 Observation of fracture modes of attachments**

Fracture mode analysis is valid but needs more detail (microscope magnification, classification criteria, observer info, quantitative results).

a-Magnification Not Specified:Magnification used for fracture observation is missing.
Suggestion: Specify the magnification or range (e.g., ×10, ×20).
b- Fracture Classification Criteria Missing:Criteria for assigning fractures to categories isn’t explained.
 Suggestion: Briefly explain how fracture types were categorized (e.g., visual percentage estimate).
c-Observer Information and Blinding Not Mentioned:No details on number of observers or blinding during analysis.
Suggestion: Clarify if one or multiple observers evaluated the samples, and if blinding was used.

d- Quantitative Reporting Needed:Fracture modes are described qualitatively, but PLOS ONE expects quantitative data.
Suggestion: Provide percentages or counts of each fracture mode per group and mention statistical tests used.

**2.6 Statistical analysis**

Statistical methods are sound but require more detail for full transparency.

a-Normality Testing Not Reported:Normal distribution claimed, but method (e.g., Shapiro-Wilk) not specified.
 Suggestion: State how normality was assessed.
b-Variance Homogeneity Not Addressed:No mention of testing (e.g., Levene’s test) for ANOVA assumptions.
Suggestion: Briefly describe variance testing.
c-Post-hoc Testing Missing:Post-hoc comparisons after ANOVA not reported.
Suggestion: Specify post-hoc test used (e.g., Tukey’s, Bonferroni).
d-Power Analysis Absent: No indication of study power or sample size justification.
Suggestion: Add a note on statistical power if possible.

**3 Results**

3.1 **Scanning Electron Microscope (SEM)**

a-The paragraph is long and a bit difficult to follow.

suggestion: a-Split it into smaller paragraphs based on different observations (e.g., healthy vs fluorotic enamel, acid etching alone, Er:YAG + acid etching).

b-Use more consistent sentence structures

b-Add quantitative or descriptive comparison if possible:

suggestion: a- For instance, describing how much rougher the fluorotic enamel is compared to healthy enamel in micrometers if data available.

b-Or describing depth/size of the pits qualitatively if measurements are not available.

c-Link the SEM findings back to bonding implications briefly:

suggestion a-Why is the honeycomb structure or enamel column dissolution important for bonding strength? One or two sentences would help the reader.

d-Magnification: The specific magnifications for each image should be stated in the figure legends.

e-Figure Presentation: Ensure scale bars are visible, and legends should better describe the morphological features.

3.2 **Shear Bonding Strength (SBS)**

a-Organization and Flow:

suggestion:The results are listed one after another without much logical flow or segmentation.

b-Clarity:It's not immediately clear what each group (A1, B1, C1, etc.) means unless the reader keeps checking Table 1.

suggestion :You can add short reminders ("Group A1: fluorotic enamel + bracket with 30s etching").

c-Better Reporting of Statistical Results:

suggestion:whenever you mention a p-value, it's better to also state the test (e.g., *one-way ANOVA*, *t-test*).

3.3 **Fracture modes of attachments**

a-How many samples were in each fracture mode (percentages or counts)? This data is missing but essential.

b-No connection to mechanical behavior**:**

Higher bond strength is usually associated with mixed fractures or cohesive fractures inside the resin. You should mention this explicitly.

c-Vague statistical reporting:

-You mention an F-value but don't state which statistical test you used (probably a chi-square test for categorical data).

-Saying "P<0.05" is fine, but mentioning whether it's "highly significant" or "statistically significant" would be better.

**4 Discussion**

a- Too long and repetitive

Suggestion : Be more concise. Avoid repeating results; focus on interpretation and meaning.

b- No clear structure

Suggestion : Organize clearly: summary of findings → comparison with past studies → mechanisms explanation → clinical implications → limitations and future work.

c- Weak linking to literature

Suggestion : Discuss how your findings compare to previous studies more clearly. Use more "According to X et al..."

d- Not enough mechanism explanation

Suggestion : Explain why Er:YAG laser + acid etching improves bonding — is it increased surface roughness? Opened prism structure?

e- Weak limitations

Suggestion : Discuss specific limitations like small sample size, in vitro setting, no

**5 Conclusion**

a- Too long and wordy

Suggestion :Make it sharper, summarized in 3 strong points maximum.

b- Repeats Discussion points

Suggestion :Focus only on final results and main message, no detail

c- Weak phrasing ("it was concluded that...")

Suggestion :Use active voice. Start directly with your conclusion statements

d- Mixes results and interpretations

Suggestion :Separate: what was found → what it means clinically.

e-No clinical recommendation

Suggestion :Add a short sentence about how your results can guide orthodontic practice

**6 References**

Your references are good — just needs technical polishing and a few updates to make it strong and professional for PLOS ONE standards.

a- Inconsistent citation style

Suggestion :Sometimes journal names are fully written, sometimes abbreviated incorrectly (e.g., "Scientific Reports" vs "Scientific reports."). Stick to PLOS ONE style (full journal name, title case).

b- Redundant references

Suggestion :You cite some similar systematic reviews twice without clarifying why (e.g., Er:YAG laser systematic reviews). Select only the most relevant or clearly explain if citing two distinct reviews.

c- Weak citation in critical points

Suggestion :When you argue important points (e.g., "prolonged acid etching improves bond strength"), you only cite 1–2 papers. You could strengthen it by citing a second or third supporting article.

d- Too few very recent references (after 2022)

Suggestion :Especially about clear aligners and enamel laser treatment — the field is advancing fast. You might want to add 1–2 more references from 2023–2024 if possible.
