# Supplementary material for: Effects of Er: YAG laser and acid etching on bond strength of clear aligner attachments to fluorotic enamel
Source: PLoS One. 2025 Aug 7;20(8):e0328937. doi: 10.1371/journal.pone.0328937 (PMC12331112; doi:10.1371/journal.pone.0328937)
Supplement: Attachment — Submitted filename: Response to Reviewers.docx [file pone.0328937.s006.docx]

**Manuscript ID:** PONE-D-25-18609 **Title:** Effects of different fluoride enamel surface preparations on the bond strength of Clear Aligner attachments **Journal:** *PLOS ONE*

**Dear Editor and Reviewers,**

We appreciate the opportunity to revise our manuscript titled " Effects of different fluoride enamel surface preparations on the bond strength of Clear Aligner attachments " and are grateful for the insightful comments provided by the reviewers. Those comments are all valuable and very helpful for revising and improving our paper, as well as providing important guiding significance for our research. In the following, we have provided detailed responses to each of the reviewers' comments. Revised portions are marked in red in the paper. Additionally, we have conducted a comprehensive revision of the entire manuscript. In this response letter, the reviewers' comments are presented in italics, and our corresponding changes and additions to the manuscript are highlighted in red text. We have tried our best to ensure that all the revisions are clear, and we hope that the revised manuscript meets the requirements for publication.

**Responds to the review’s comments:**

**Reviewer #1：**

**Comment 1:** *Yes, the manuscript is technically sound. The study is well-designed, with appropriate sample sizes, controls, and methodology. The data—including SEM observations, shear bond strength (SBS) measurements, and fracture analysis—clearly support the conclusions. The findings are statistically significant and align well with the study’s stated objectives.*

**Response 1:** We sincerely thank the reviewer for their positive assessment of our study’s rigor and validity.

**Comment 2:** *Yes, the statistical analysis is appropriate and rigorous. One-way ANOVA, t-tests, and chi-square tests were used correctly. Significance levels and SBS results are clearly reported. However, details on normality testing, post hoc comparisons, and power analysis are missing and should be added for transparency.*

**Response 2:** We sincerely appreciate your thoughtful review and positive assessment of our statistical approach. Thank you for highlighting the need for greater transparency regarding normality testing, post hoc comparisons, and power analysis—we fully agree these elements strengthen methodological rigor. In response to your valuable suggestion, we have added the following details to the Statistical Analysis section **(Section 2.6, Page 9)** in the revised manuscript.

a) Normality Testing: Data distribution was confirmed via Shapiro-Wilk tests (p > 0.05 for all groups), supporting the use of parametric tests.
b) Post Hoc Comparisons: Following significant ANOVA results, Tukey’s HSD test was applied for pairwise group comparisons to control Type I error.
c) Power Analysis: A power analysis (GPower 3.1; α=0.05, power=0.85, effect size f=0.55) indicated a minimum requirement of 10 specimens per subgroup. This aligns with precedents for bond strength studies [20, 27, 45].

[20] Ng'ang'a PM, Øgaard B, Cruz R, Chindia ML, Aasrum E. Tensile strength of orthodontic brackets bonded directly to fluorotic and nonfluorotic teeth: An in vitro comparative study. American Journal of Orthodontics and Dentofacial Orthopedics. 1992;102(3):244-50. doi: https://doi.org/10.1016/S0889-5406(05)81059-5.

[27] Bassir MM, Rezvani MB, Ghomsheh ET, Hosseini ZM. Effect of Different Surface Treatments on Microtensile Bond Strength of Composite Resin to Normal and Fluorotic Enamel After Microabrasion. Journal of Dentistry (Tehran, Iran). 2016;13(6):431-7. Epub 2017/03/01. PubMed PMID: 28243305; PubMed Central PMCID: PMCPMC5318500.

[45] Kamran MA, Asiri AM, Alfaifi AMA, Almukawwi AHA, Mughaddi Alwadai J, Alqahtani SJ. Fluoride-Activated Via Er:YAG, Diode, and Femtosecond Lasers for Reversing Bleached Enamel for Improved Orthodontic Bonding. Photobiomodulation, Photomedicine, and Laser Surgery. 2025. Epub 2025/06/09. doi: https://doi.org/10.1089/photob.2025.0024. PubMed PMID: 40485293.

**The revised statistical analysis now reads:** Statistical analysis was performed using the SPSS 20.0 (Chicago, IL, USA) program. All data were expressed by means ± standard deviation (‾x ± s). Normality was confirmed by Shapiro-Wilk tests (α = 0.05), and variance homogeneity was verified with Levene's test before parametric analyses. Between-group variation was assessed using a t-test or one-way ANOVA (with Tukey's HSD post-hoc tests for significant effects). Power analysis (1−β = 0.85, α = 0.05) determined the adequate sample size. All tests were performed at a 95% conﬁdence. A P-value≤0.05 was considered significant. (ns *P* > 0.05, * *P* <0.05, ** *P* < 0.01, *** *P* < 0.001, **** *P* < 0.0001) **(Page 9, line 158-164)**

**Comment 3:** *Yes, the authors have made all data fully available. Raw SBS values, SEM data, and fracture classifications are included in the manuscript. There are no restrictions on access, fulfilling PLOS ONE's data sharing requirements.*

**Response 3:** We are truly grateful for your acknowledgment of our commitment to open science and data transparency. Thank you for confirming that our data sharing practices align with PLOS ONE's rigorous standards—this validation is deeply encouraging to our team.

**Comment 4:** *The manuscript is generally understandable but needs revision for grammar, clarity, and consistency. Issues include awkward phrasing, typographical errors, and inconsistent terminology. Language editing is recommended to meet publication standards.*

**Response 4:** We sincerely thank you for your careful reading and constructive feedback on the manuscript’s language quality. In response to your valuable suggestion, we have taken the following comprehensive actions:

a) The full manuscript was rigorously processed through Grammarly Premium (Academic setting) to correct grammatical and syntactic errors, eliminate typographical mistakes， simplify complex sentences, and ensure tense consistency (e.g., unified past tense for methods)
b) To standardize terminology, we created a master terminology list (e.g., "shear bond strength (SBS)" consistently used) and manually verified all technical terms (e.g., "fluorotic enamel" vs. "fluorosed enamel", "enamel column" vs. "enamel rod"). We revised 13 instances of ambiguous phrasing in the Discussion
c) Partial examples of key Improvements: In the Methods section, "Teeth was etched..." was changed to "Enamel surfaces were etched...". In the Results section, "...can be seen" was revised to "...was evident".
d) We conducted three rounds of team proofreading focusing on subject-verb agreement, article usage (a/an/the), and parallel structure in lists.

Your guidance has significantly improved the manuscript’s readability. We have attached a revised copy showing all linguistic corrections in red. If there are any remaining issues we may have overlooked, we would appreciate the opportunity to refine further.

**Reviewer #2:**

*The manuscript (PONE-D-25-18609) investigates whether the optimum combination of Er: YAG laser and acid etching improves the bonding ability of the attachment on fluorotic enamel. The authors aim to address optimizing enamel surface preparation methods to enhance the bond strength of clear aligner attachments in teeth affected by moderate dental fluorosis. Below are my comments and suggestions for improvement:*

**Comment 1:** *Introduction Section: The manuscript contains numerous grammatical errors, awkward phrasing, and non-native usage. The introduction needs revision for grammar, sentence structure, and overall fluency.*

**Response 1:** We sincerely thank you for your meticulous critique. Your guidance has been instrumental in elevating the scholarly rigor of our work. According to your valuable suggestion, we have taken the following comprehensive actions:

a) Unrelated laser studies (periodontitis, bracket re-bonding) have been moved to the Discussion section. **(Page 17, line 282-284)**
b) Transition sentences have been added to connect the prevalence of fluorosis, treatment gaps, and the rationale for the study:

“Moreover, the demand for orthodontic treatment is constantly rising. However, managing fluorotic enamel poses unique challenges in orthodontic practice, particularly during bracket bonding procedures **(Page 3, line 49-51)**. Conventional acid etching techniques, while effective on normal enamel, are inadequate for fluorotic surfaces due to their altered chemical composition and porosity characteristics. Therefore, this study aimed to…” **(Page 3, line 55-59)**

c) The study aim has been repositioned to the end of the second paragraph:

" Therefore, this study aimed to investigate whether combining Er: YAG laser with acid etching could enhance the bonding strength of clear aligner attachments on fluorotic enamel compared to conventional acid etching alone." **(Page 3, line 57-59)**

**Comment 2**: *Materials and Methods:
•Expand the discussion on the mechanisms of improved bonding with Er: YAG laser treatment. Did the authors vary the laser parameters? How can these parameters affect the etching process?
•Although the description could use more clarity and detail, the etching process is generally understandable. Indicate, for instance, if the teeth were air-dried using compressed air free of oil or blotted, and if the enamel, dentin, or both were etched. Additionally, the 5-second rinse might be regarded as brief; standard procedure typically suggests 10–15 seconds to guarantee total etchant removal.*

**Response 2:** We sincerely appreciate the reviewer’s insightful request to enhance the mechanistic discussion and clarify our parameter selection process. In response to your suggestion, we have significantly expanded the Discussion section **(Page 17, line 289-295)** to explain the mechanisms behind improved bonding with Er: YAG laser treatment.

Regarding the change in laser parameters and its influence on the etching process, this is a critical question you raised. In this study, we used a specific set of laser parameter combinations based on previous literature reports and our preliminary experiments. While laser parameters have been provided, specific details, such as pulse duration, are absent. **In this regard, we have included the missing details in the Materials and Methods (Page 5, line 100-102) as follows:** 2940nm, 100mJ energy, 30Hz frequency, pulse duration 250 μs, 6/8 water volume, 1.3*14 mm working tip, 2mm distance from the working tip to the dental surface, irradiating 10s.

In addition, we fully agree that varying laser parameters (such as energy, frequency, pulse duration, water cooling, and irradiation time) will significantly affect the etching effect. We acknowledge that the Materials and Methods section lacks discussion on how these parameters specifically affect the etching process. **To briefly explain the rationale behind selecting these specific parameters, we have added the following to the discussion section:** We chose a mode specifically designed for dental hard tissue, particularly hypoplastic enamel. The energy output was limited to 100 mJ (30Hz frequency) to prevent structural damage while effectively altering the enamel surface [44]. A 250 μs ultra-short pulse duration was utilized to significantly reduce the thermal impact on the pulp [45]. Finally, based on SEM results from Group C, a 10-second irradiation produced an ideal honeycomb surface morphology suitable for resin bonding, while extended irradiation times caused structural damage. **(Page 17, line 299-304)**

About the details of the etching process, we modified the content of the manuscript according to your opinion. **The modified content is as follows:**

“…the etched enamel surfaces were rinsed with water for 10 seconds and dried with clean air (oil-free and water-free) for 3 seconds.” **(Page 6, line 103-104)**

“The enamel samples in each group were acid etched using 37% phosphoric acid for 30 seconds (Heraeus Kulze, Germany), then rinsed with running water for 10 seconds and dried with clean air (oil-free and water-free) for 3 seconds.” **(Page 6, line 106-108)**

We are profoundly grateful for your guidance in strengthening this critical aspect. Should any mechanistic details require further clarification, we welcome the opportunity to refine them.

**Comment 3:** *Results: The results are well written, but some of the figures need better labels and explanations.*

**Response 3:** We are profoundly grateful for this constructive feedback and wholeheartedly agree that visual clarity is essential for data interpretation. We sincerely apologize for any deficiencies in our original figures and have implemented comprehensive revisions to all graphical elements. **Below, we detail the specific improvements made and present the partially** **modified figure (Page 11-12, line 183-199):**

a) improved resolution for each figure in the manuscript (not less than 300 dpi, meeting PLOS ONE).
b) added arrows to each picture for illustration purposes.
c) added scale bars to the caption.
d) added description of morphological features to the caption.

|  |  |
| --- | --- |
|  |  |

**Figure 1.** Micro appearance of the enamel surface. (a) healthy teeth group: smooth and flat enamel surface (magnification ×3000, 50μm); (b) healthy teeth group: smooth and flat enamel surface (magnification ×50000, 3μm); (c) dental fluorosis group: rough enamel surface, exhibiting varying degrees of pit defects (magnification ×3000, 50μm); (d) dental fluorosis group: rough enamel surface, exhibiting varying degrees of pit defects (magnification ×50000, 3μm).

|  |  |  |
| --- | --- | --- |
|  |  |  |

**Figure 2.** Micro appearance of the enamel surface of dental fluorosis after different interventions: (a) etching for 30s group: incomplete etching of the enamel surface was observed (arrow); (b) etching for 60s group: a uniform depression around the enamel rod was evident, displaying an irregular honeycomb shape (arrow); (c) etching for 90s group: the dissolution of the enamel rod along with a smear layer on the enamel surface was visible (arrow); (d) etching for 30s after Er: YAG laser irradiating group: the enamel surface was pitted and the boundary around the enamel rod was clear (arrow); (e) etching for 60s after Er: YAG laser irradiating group: the enamel surface exhibited a uniform fish scale-like texture, with the center of the enamel rod being concave and the edge evident (arrow); (f) etching for 90s after Er: YAG laser irradiating group: irregular pits appeared on the enamel surface with enamel dissolution, and the boundary between the enamel rods became unclear (arrow). (all magnification ×3000, 50μm).

Additionally, we have reorganized the structure of the Results paragraphs. We have split it into smaller paragraphs based on different observations and used more consistent sentence structures. **The partially changed paragraphs are as follows (Section 3.1, Page 10, line 167-182)**:

Surface of Enamel: Under SEM, the surface of healthy enamel appeared smooth and flat, while that of fluorotic enamel seemed rough, exhibiting varying degrees of pit defects (Fig. 1). These structural differences may influence the bonding effectiveness, as the irregular surface can impact the penetration of the adhesive.

Acid Etching Effects: Phosphoric acid etching produced time-dependent structural changes: ①30s etching: incomplete etching of the enamel surface was observed (Fig. 2A); ②60s etching: a uniform depression around the enamel rod was evident, displaying an irregular honeycomb shape (Fig. 2B); this micro-retentive pattern is crucial for resin tag penetration and enhancing bonding strength. ③90s etching: the dissolution of the enamel rod along with a smear layer on the enamel surface was visible (Fig. 2C), which may compromise mechanical interlocking.

Er: YAG Laser-Assisted Acid Etching: The combined laser-acid treatment produced distinct effects:①Er: YAG + 30s etching: the enamel surface was pitted and the boundary around the enamel rod was clear (Fig. 2D); ②Er: YAG + 60s etching: the enamel surface exhibited a uniform fish scale-like texture, with the center of the enamel rod being concave and the edge evident (Fig. 2E); ③Er: YAG + 90s etching: irregular pits appeared on the enamel surface with enamel dissolution, and the boundary between the enamel rods became unclear (Fig. 2F).

**Comment 4:** *Expand the discussion to explain why laser-etched enamel might improve bond strength. Some procedural details and justifications are missing.*

**Response 4:** We sincerely thank the reviewer for this insightful suggestion. We fully agree that elucidating the mechanism of Er: YAG laser etching is critical for interpreting our findings and have significantly expanded the Discussion section to address this point.

**The added mechanism explanation now reads:** The Er: YAG laser (2940 nm) is highly absorbed by water and hydroxyapatite in enamel, resulting in the micro-explosion phenomenon. This effect effectively and selectively removes areas of lesions or mineralization abnormalities, resulting in a clean, rough, yet not excessively destructive honeycomb structure on the surface of fluorotic enamel. This not only provides the basis for the micromechanical interlocking structure and increases the bonding area, but also removes the smear layer and exposes a more reactive fresh enamel, facilitating resin penetration and chemical bond formation. The SEM results from group C observed in this study support this mechanism. **(Page 17, line 289-296)**

**We have added the following critical technical justifications to the Discussion section:** Various studies have examined laser etching with different irradiation settings. In this study, the laser parameters were configured according to the device's instructions to ensure the safety and efficacy of fluorosis enamel treatment. We chose a mode specifically designed for dental hard tissue, particularly hypoplastic enamel. The energy output was limited to 100 mJ (30Hz frequency) to prevent structural damage while effectively altering the enamel surface [44]. A 250 μs ultra-short pulse duration was utilized to significantly reduce the thermal impact on the pulp [45]. Finally, based on SEM results from Group C, a 10-second irradiation produced an ideal honeycomb surface morphology suitable for resin bonding, while extended irradiation times caused structural damage. **(Page 17-18, line 297-304)**

**Reviewer #3:**

*Dear author(s),
Thanks for sharing your work with us, the followings are the revisions and suggested comments that are needed to be taken in consideration:*
**Comment 1***: The manuscript's quality and data presentation are acceptable and important for clinicians and even patients.*

**Response 1:** We are profoundly grateful for your generous assessment of our work's clinical significance and scientific contribution. Your recognition that our findings hold value "for clinicians and even patients" is deeply meaningful to our team—this dual impact on both dental practice and patient outcomes represents the core aspiration of our research.

**Comment 2***: The manuscript advances our understanding of enamel surface preparations on the bond strength of Clear Aligner attachments and the combination of Er: YAG laser and acid etching improve the bonding ability of clear aligner attachment on the surface of dental fluorosis.*

**Response 2:** We are sincerely honored that you consider our study to advance understanding of enamel surface preparations for clear aligner therapy, particularly regarding Er: YAG laser-enhanced bonding in fluorotic cases.

**Comment** **3**: *The title should be rewritten to be more precise and explanatory.*

**Response 3:** We sincerely thank you for this valuable suggestion to refine our manuscript title. We agree that the original title did not fully capture the precision and clinical relevance of our findings, and we deeply appreciate your guidance in enhancing its explanatory power.

**The revised title now reads:** Effects of Er: YAG Laser and Acid Etching on Bond Strength of Clear Aligner Attachments to Fluorotic Enamel **(Page 1, line 1-2)**

**Comment 4**: *Make the abstract more informative and it should represent the article's substance and be no more than 250 words long.*

**Response 4:** We sincerely appreciate your insightful guidance on refining the abstract to enhance its clarity and conciseness while ensuring it fully captures the essence of our study. In response to your suggestion, we have:

a) Reduced the length from 245 to 164 words (meeting PLOS ONE).
b) Restructured the content into clearer sections (Background, Methods, Results, Conclusion).
c) Removed excessive technical minutiae: Deleted fracture mode statistics.
d) Enhanced language precision: Corrected grammatical errors (e.g., verb tenses) and simplified lengthy sentences.
e) Sharpened focus on primary discovery: Emphasized the synergy between Er: YAG and acid etching and highlighted the SBS increase compared to controls.

**The revised abstract now reads (Page 1, line 15-29):**

**Background:** There is a lack of research on the bonding ability of attachments to fluorosis enamel. This study evaluates Er: YAG laser-assisted acid etching as a potential optimization protocol.

**Methods:** Twenty healthy teeth and ninety fluorotic teeth (Thylstrup-Fejerskov Index=4) were divided into a control group (healthy enamel + acid etching) and a fluorotic group (acid etching 30/60/90 seconds vs. Er: YAG laser + acid etching). The enamel surface was analyzed using scanning electron microscopy, and the shear bond strength (SBS) of each group (n = 10/group) was tested.

**Results:** Attachments exhibited higher SBS than brackets (p<0.01). Laser-acid etching enhanced SBS compared to acid etching alone (p<0.01). Laser-treated surfaces exhibited predominantly mixed fracture and resin cohesion fractures, in contrast to adhesive interface fractures and mixed fractures in the acid-only groups.

**Conclusion:** Er: YAG laser (100mJ/30Hz) with 60 seconds acid etching achieves excellent bonding for fluorotic enamel attachments, restoring adhesion to healthy enamel levels while preventing over-etching damage. This protocol shows clinical potential for bonding fluorotic enamel.

**Comment** **5**: *Include four to six keywords that are relevant to the manuscript but not stated in the title.*

**Response 5:** We sincerely thank you for this thoughtful suggestion to enhance the manuscript’s discoverability through targeted keywords. We deeply appreciate your guidance in ensuring our work reaches the appropriate clinical and academic audiences—this refinement is invaluable for maximizing its impact.

**The revised keywords now read:** Dental fluorosis, Orthodontic bracket, Clear aligner attachment, Shear bond strength, Er: YAG laser, Acid etching, Surface treatment. **(Page 2, line 31-32)**

**Comment 6**: *Additional paragraphs to introduce further studies on other applications of laser in dental and surgical management of various clinical entities .
Suggested references:
Aldelaimi, A.A., Ahmed, R.F., Enezei, H.H., Aldelaimi, T.N.. Gummy smile esthetic correction with 940 nm diode laser. International Medical Journal 26(6): 513 - 515 , 2019
Aldelaimi TN, Khalil AA. Clinical Application of Diode Laser (980 nm) in Maxillofacial Surgical Procedures. J Craniofac Surg. 2015;26(4):1220-1223. doi:10.1097/SCS.0000000000001727*

**Response 6:** Thank you for generously sharing these highly relevant references—your expertise has guided us to strengthen the clinical context of our work, and we sincerely appreciate your commitment to enhancing the manuscript’s interdisciplinary value.

We integrated your recommended references and added new content to the Discussion:

**The added content now reads (Page 17, line 280-282):** Nowadays, various lasers are utilized in dental treatments, such as gingivectomy for correcting a gummy smile [34,35], and maxillofacial surgery due to their effectiveness in hemostasis and reducing pain and swelling [36].

[34] Aldelaimi T, Aldelaimi A, Enezei H, Faisal R. Gummy Smile Esthetic Correction with 940 nm Diode Laser. International Medical Journal (1994). 2019;26:513-5.

[35] Silva DFB, de Freitas GA, Leite L, Lucena KCR, de Castro Gomes DQ. Gingivectomy with high-power laser for correction of the gummy smile resulting from altered passive eruption-a case series. Lasers in Medical Science. 2022;37(7):2999-3009. Epub 2022/06/07. doi: https://doi.org/10.1007/s10103-022-03586-0. PubMed PMID: 35661938.

[36] Aldelaimi TN, Khalil AA. Clinical Application of Diode Laser (980 nm) in Maxillofacial Surgical Procedures. The Journal of Craniofacial Surgery. 2015;26(4):1220-3. Epub 2015/06/17. doi: https://doi.org/10.1097/scs.0000000000001727. PubMed PMID: 26080161.

**Comment 7**: *The photos (Figures) are omitted and NOT available within text. Care should be taken to improve resolution and contrast for each figure in the manuscript and arrows to each picture for illustration purposes.*

**Response 7:** We sincerely apologize for this technical oversight and deeply appreciate your vigilance in identifying it. We confirm this was caused by an unexpected file conversion error during submission, likely due to embedded image layers. We have now re-uploaded all figures to comply with journal specifications. All figures have been re-exported as 600 dpi TIFFs and directly embedded in the manuscript text. **The partially modified pictures are as follows:**

|  |  |
| --- | --- |
|  |  |

|  |  |  |
| --- | --- | --- |
|  |  |  |

**Comment 8**: *Authors should check for writing and typing errors.*

**Response 8:** We sincerely thank the reviewer for highlighting this critical issue. We deeply regret that language imperfections remained in our initial submission and recognize that clear communication is essential for scientific rigor. To address this comprehensively, we have thoroughly processed the full manuscript through Grammarly Premium (Academic setting) to correct grammatical and syntactic errors, eliminate typographical mistakes, simplify complex sentences, and ensure tense consistency (e.g., unified past tense for methods). The revised manuscript now passes automated checks with zero critical errors in Grammarly Premium and zero spelling mistakes via Microsoft Word Editor. In addition, all co-authors performed three rounds of line-by-line reviews focusing on grammar, syntax, typographical errors, and consistent use of technical terminology (e.g., "shear bond strength" vs. "bonding strength").

We have attached a revised copy showing all linguistic corrections in red. We are profoundly grateful for the reviewer’s vigilance in identifying this shortcoming. Our team has taken this critique seriously, and we hope the revised manuscript now meets the journal's linguistic standards. If there are any remaining issues we may have overlooked, we would appreciate the opportunity to refine further.

**Comment 9**: *The statements in discussion are acceptable but few paragraphs about the justification of your findings and comparison with other recent relevant studies.*

**Response 9:** We are deeply grateful for this invaluable suggestion. We have now comprehensively restructured the Discussion following your guidance, with key improvements detailed below：

a) reorganized structure to "summary of findings → comparison with past studies → mechanisms explanation → clinical implications → limitations and future work".
b) **added direct literature comparisons as follows**:

“Bassir et al. found that fluorosis reduced the bonding performance of enamel and had nothing to do with the properties of bonding materials (metal or resin) [27], which was consistent with our hypothesis.” **(Section 4.2, Lines 248-250)**

“According to Gorler et al.’s study [28], the bonding strength of 6 - 8MPa can meet the clinical needs of the bonding strength of brackets in fixed orthodontics, which is close to the results of our study, 7.12- 9.72MPa.” **(Section 4.2, Lines 252-254)**

“Alavi et al.’s study showed that laser etching at 100 mJ energy produced bond strength similar to acid etching [42]. In contrast, Sallam et al. [43] found that there is no significant difference in SBS between Er: YAG laser and acid etching, respectively. Kuhn et al. argue that the Er: YAG laser may cause damage to resin composites, which could limit the use of laser irradiation [44]. However, their study focused on healthy enamel, a significant difference considering the lower reactivity of fluorotic enamel.” **(Section 4.2, Lines 284-289)**

c) **added the following to the discussion section to briefly explain the justification of the protocol** **(Section 4.3, Lines 299-304):** We chose a mode specifically designed for dental hard tissue, particularly hypoplastic enamel. The energy output was limited to 100 mJ (30Hz frequency) to prevent structural damage while effectively altering the enamel surface [44]. A 250 μs ultra-short pulse duration was utilized to significantly reduce the thermal impact on the pulp [45]. Finally, based on SEM results from Group C, a 10-second irradiation produced an ideal honeycomb surface morphology suitable for resin bonding, while extended irradiation times caused structural damage.

**Comment 10:** *Only include current references in the reference list and remove outdated ones.*

**Response 10:** We would like to express our sincere gratitude for this valuable guidance, which has significantly enhanced the academic foundation of our work. In response to your suggestion, we have updated the references systematically through the following processes:

First, we identified and removed five outdated publications, such as Nalcaci et al. 2015, Mendes et al. 2014, and Lihua et al. 2013.

Secondly, we added seven updated publications (Scribante A et al. 2022, Silva DFB et al. 2022, and Kamran MA et al. 2025, etc.), which are highlighted in the revised version.

Thirdly, after careful consideration, we retained some seminal works: Limeback' s 1994 study (authoritative interpretation of the characteristics of fluoride enamel), Castilho's 2009 perceptions (no modern equivalent exists), and Ng'ang'a' s 1992 comparative study (published in Q1 authoritative journals, with 23 citations), etc.

**Other changes:**

a) Abstract section: We reorganized the abstract into ‘Background, Methods, Results, and Conclusions’ to make it more closely aligned with the article content and improve readability;
b) Introduction section: Added sentences to enhance the flow between paragraphs, guiding readers from the problem to the current solutions, gaps, and study aim.
c) Methods Section: Added a description of the blinded method.
d) Discussion Section: Specified the limitations of the study, including limited sample size, lack of oral environment, and testing of only a single laser parameter.
e) Conclusion Section: Simplified the conclusion into three points and added clinical significance.

We tried our best to improve the manuscript and made some changes marked in red in the revised paper, which will not influence the content and framework of the paper. We also refined the language of the manuscript, and the revised manuscript is better than before! We sincerely appreciate the Editors’ and Reviewers' dedicated work and hope the correction will receive approval. Best regards!
